# Enhanced Nanoparticle Sensing by Sagnac–Fizeau Shift in a Microcavity Based on Exceptional Surfaces

**DOI:** 10.3390/s25196055

**Published:** 2025-10-02

**Authors:** Qingde Yang, Peixin Chen, Tonghua Hu, Shuo Jiang

**Affiliations:** Hangzhou Institute for Advanced Study, University of Chinese Academy of Sciences, Hangzhou 310024, China

**Keywords:** exceptional surface, Sagnac-Fizeau shift, nanoparticle sensing

## Abstract

The exceptional surface (ES) in non-Hermitian physics has attracted much attention due to its strong robustness and enhanced frequency splitting in the sensing field. However, the detection limit of the ES-based sensing structure is still limited by the mode linewidth in the optical microcavity. In this paper, we demonstrate that Sagnac–Fizeau shift in a microcavity based on an ES separates the dark mode from the bright mode, further enhancing the frequency splitting in the transmission spectrum. Moreover, a strategy for manipulating spectral line shape is realized by the phase in the reflection loop. Compared with the traditional ES-based sensing structure, the proposed nanoparticle sensing mechanism significantly reduces the detection limit for weak perturbations. This work will contribute to the development of high-precision nanoparticle sensors.

## 1. Introduction

Exceptional points (EPs) with rich physical properties are non-Hermitian spectral degeneracies, where two or more eigenvalues and their corresponding eigenvectors coalesce [1,2,3,4,5,6,7,8]. The reduction in the dimensionality of the system’s eigenvectors makes EPs highly sensitive to any weak perturbations, which can enhance the frequency splitting response in sensing fields [9,10,11,12,13,14,15,16,17]. Nanoparticle sensing has become a cutting-edge research hotspot in the current sensing field due to the unique advantage of its nanoscale resolution [18,19,20,21]. As an open system, the optical microcavity provides an important platform for achieving the nanoparticle sensing mechanism based on EPs. The frequency splitting response of EP-enhanced nanoparticle sensing has been observed in parity–time (PT) symmetric microcavities [18], anti-parity–time (anti-PT) symmetric microcavities [19,20], and chiral microcavities [21]. The realization of isolated EPs in these systems requires accurate control of experimental parameters, which hinders the practical application of EP-based nanoparticle sensors. An exceptional surface (ES) consists of a series of EPs extending isolated points to a two-dimensional hypersurface [22]. It reduces the requirements of some parameters and has strong resistance to manufacturing errors and environmental uncertainties. On the other hand, an ES has the same enhancement effect as EPs in terms of frequency splitting response [23,24]. The frequency splitting enhancement and robustness of an ES in optical microcavities have been experimentally verified [25,26,27].

However, ES-based nanoparticle sensors are limited by the quality factor (Q factor) of the optical microcavity meaning that they cannot obtain distinguishable frequency splitting in the spectrum for small-sized nanoparticles. To further enhance the detection limit of the ES-based nanoparticle sensor, methods such as reducing losses in the optical microcavity can be adopted. In addition, utilizing the Sagnac–Fizeau shift in optical microcavities to increase the frequency splitting response can help improve the detection limit of nanoparticles [28]. Combining the Sagnac–Fizeau shift with a microcavity based on an ES may further enhance the frequency splitting response and improve the detection limit of the ES-based nanoparticle sensors. Unfortunately, the Sagnac–Fizeau-shift-enhancing nanoparticles in the sensing structure based on an ES have not been studied.

In this paper, we theoretically investigate the mechanism of enhanced nanoparticle sensing by the Sagnac–Fizeau shift in an ES-based optical microcavity. This mechanism diverges fundamentally from our prior work in both principle and structure [29], yielding more intriguing observations. Under unidirectional coupling between two optical modes, the Sagnac–Fizeau shift caused by rotating microcavities results in two eigenvalues of the structure existing in bright and dark modes, respectively. Also, a strategy to regulate the spectral line shape through the phase in the reflection loop is proposed and analyzed. Compared with the traditional ES-based nanoparticle sensors, the proposed nanoparticle sensing mechanism makes the frequency splitting easier to distinguish, which improves the detection limit for small-sized nanoparticles.

## 2. Theoretical Models

**Figure 1:**
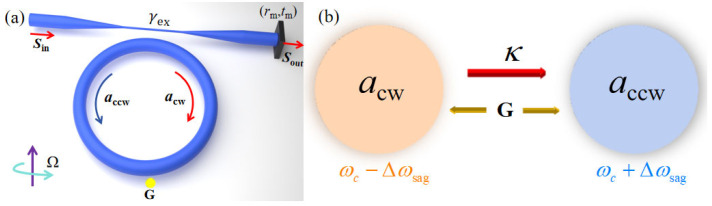
(**a**) Schematic diagram of the nanoparticle sensing mechanism based on an ES with a spinning microcavity. The coupling rate and loss caused by a nanoparticle can be seen as a perturbation G. (**b**) A schematic of the interaction between mode *a*_cw_ and mode *a*_ccw_. Here, $\kappa =-it_{0}\gamma_{\mathrm{ex}}e^{i\varphi }$, $t_{0}$, and φ are the transmission amplitude and phase accumulation in the reflection, respectively.

Figure 1a presents the schematic diagram of using the Sagnac–Fizeau-shift-enhanced nanoparticle sensing mechanism in an ES-based microcavity. The proposed structure is composed of a passive microcavity (diameter *d* = 1000 μm) and introduces input (output) light field *S*_in_ (*S*_out_) through a tapered fiber placed on the side of the microcavity. The input light (with a wavelength λ of 1550 nm) enters from the left end of the tapered fiber and is coupled (coupling coefficient $\gamma_{\mathrm{ex}}$) by evanescent wave into the optical microcavity to form the clockwise mode *a*_cw_. The right end of the conical fiber is connected with a partially reflective mirror (reflection coefficient *r*_m_ and transmission coefficient *t*_m_). The mode *a*_cw_ couples out of the microcavity, and partial reflection excites the counterclockwise mode *a*_ccw_. Thus, the unidirectional coupling of mode *a*_cw_ and mode *a*_ccw_ within the microcavity is achieved (with a coupling strength κ), enabling the sensing structure to operate on an ES. Here, the microcavity starts rotating at an angular velocity Ω, the tapered fiber is fixed, and the position between the two is self-adjusted, which can result in stable microcavity–fiber coupling. This has been proven feasible in experiments [30]. When a nanoparticle with a perturbation strength of G appears around the evanescent field of the spinning microcavity, it causes a bidirectional coupling between the mode *a*_cw_ and mode *a*_ccw_ in Figure 1b.

The mode *a*_cw_ and mode *a*_ccw_ in a spinning microcavity undergo the rotation-induced Sagnac–Fizeau shift that can be expressed as [28,31]

(1) $$\Delta \omega_{\mathrm{sag}}=\frac{nd{\Omega \omega }_{c}}{2c}\left(1-\frac{1}{n^{2}}-\frac{\lambda }{n}\frac{dn}{d\lambda }\right)$$

where n is the refractive index of the microcavity, $\omega_{c}$ is the resonant frequency of a stationary microcavity, and c is the speed of light. The dispersion term dn/dλ is relatively small in typical materials and can be ignored [28].

When the nanoparticle is near the microcavity, the mode evolution of the ES-based sensing structure in the rotating state can be described by

(2) $$\begin{array}{l} \frac{da_{\mathrm{cw}}}{dt}=\left(-i\omega_{c}+i\omega_{\mathrm{sag}}-\gamma_{t}-iG\right)a_{\mathrm{cw}}-iGa_{\mathrm{ccw}}-i\sqrt{\gamma_{\mathrm{ex}}}s_{\mathrm{in}},\\ \frac{da_{\mathrm{ccw}}}{dt}=\left(-i\omega_{c}-i\omega_{\mathrm{sag}}-\gamma_{t}-iG\right)a_{\mathrm{ccw}}-i\kappa a_{\mathrm{cw}}-iGa_{\mathrm{cw}}-i\sqrt{\gamma_{\mathrm{ex}}}e^{i\phi }s_{\mathrm{in}} \end{array}$$

where $\gamma_{t}=\left(\gamma_{0}+\gamma_{\mathrm{ex}}\right)/2$ is the total loss of the microcavity; $\gamma_{0}$ and $\gamma_{\mathrm{ex}}$ are the intrinsic loss and coupling loss of the microcavity, respectively. $G=g-{i\gamma }_{n}$ is the complex perturbation induced by the nanoparticle, g=−Reαf(r→)2ωc/2Vm is the coupling rate between mode *a*_cw_ and mode *a*_ccw_ because of the nanoparticle, $\alpha =4\pi R^{3}\varepsilon_{l}\left(\varepsilon_{p}-\varepsilon_{l}\right)/\left(\varepsilon_{p}+2\varepsilon_{l}\right)$ is the polarizability of the nanoparticle obtained from the Wigner–Weisskopf semi-QED (quantum electrodynamics) treatment, R denotes the radius of the nanoparticle, and $\varepsilon_{p}\left(\varepsilon_{l}\right)$ represents the relative permittivity of the nanoparticle (the environment medium). $f_{\left(\vec{r}\right)}^{2}$ is the microcavity mode function, $V_{m}$ denotes the mode volume of the microcavity, and $\gamma_{n}$ is the loss caused by scattering and absorption of the nanoparticle. The explanations of all symbols can be found in the Appendix A.

The two eigenfrequencies of the sensing structure can be derived as

(3) $$\omega_{1,2}=\omega_{c}-i\gamma_{t}+G\pm \sqrt{G\kappa +G^{2}+\omega_{\mathrm{sag}}^{2}}$$

In this case, the two initial eigenfrequencies of the proposed sensing structure are no longer degenerate, unlike the traditional ES-based structure, but are related to the rotation-induced Sagnac–Fizeau shift. The perturbation G leads to further splitting of the eigenfrequencies. The eigenfrequency splitting of the proposed sensing structure can be evaluated by monitoring the transmission spectral lines. According to the input–output relationship, the amplitude of the output light is written as

(4) $$s_{\mathrm{out}}=\left(1-r_{m}\right)\left(s_{\mathrm{in}}-i\sqrt{\gamma_{\mathrm{ex}}}a_{\mathrm{cw}}\right)$$

Thus, the corresponding transmission spectrum is $T={\left|s_{\mathrm{out}}/s_{\mathrm{in}}\right|}^{2}$.

## 3. Results and Discussion

### 3.1. Enhanced Frequency Splitting in the ES-Based Structure

The two eigenvalues of the proposed nanoparticle sensing structure are closely related to the detection limit of the sensor, which depends on the unidirectional coupling strength κ and the angular velocity Ω, and the frequency splitting $\Delta \omega =\omega_{1}-\omega_{2}$ from Equation (3) can be analyzed in detail.

When κ=0 and Ω=0, the sensing structure is working at diabolic points (DPs), the two eigenvalues are degenerate, and the frequency splitting is

(5) $$\Delta \omega_{\mathrm{DP}}=2G$$

**Figure 2:**
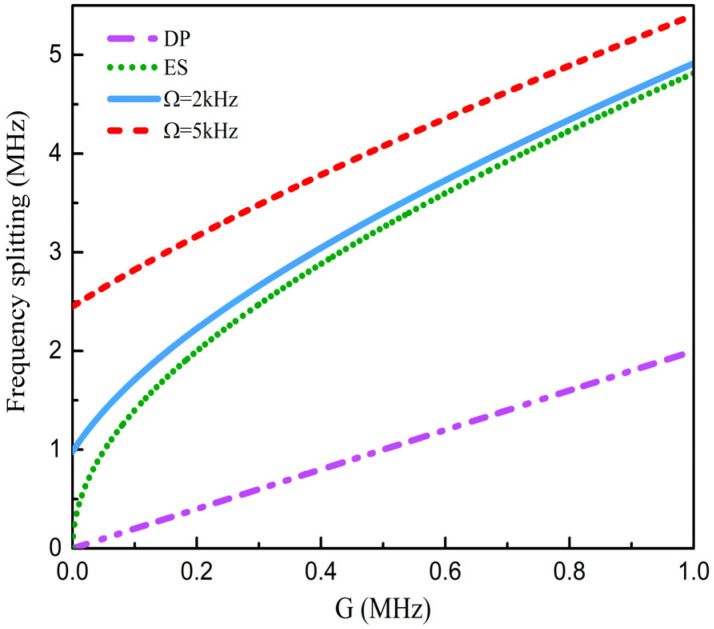
Comparison of frequency splitting of sensing structures based on different sensing mechanisms. When the frequency splitting is zero, it indicates that the two initial eigenfrequencies are degenerate. Here, $\gamma_{0}=1.37\;\mathrm{MHz}$ and $\gamma_{\mathrm{ex}}=6.85\;\mathrm{MHz}$.

The eigenvalue splitting of the structure presents a linear change with the perturbation G, as shown by the purple dashed line in Figure 2.

When κ>0 and Ω=0, the two eigenvalues of the sensing structure are initially in a degenerate state. So the sensing structure operates at an ES, and the frequency splitting can be expressed as

(6) $$\Delta \omega_{\mathrm{ES}}=2\sqrt{G\kappa +G^{2}}$$

The frequency splitting of the ES-based structure diverges with the square root of the perturbation G (the green dotted line in Figure 2), which enhances the frequency splitting under weak perturbations.

When κ>0 and Ω>0, the initial eigenvalues of the sensing structure are no longer degenerate, and the frequency splitting can be expressed as

(7) $$\Delta \omega =2\sqrt{G\kappa +G^{2}+\Delta \omega_{\mathrm{sag}}^{2}}$$

It can be seen from Equation (7) that the magnitude of the angular velocity Ω directly affects the variation in frequency splitting. Unlike the nanoparticle sensors based on a spinning microcavity, the frequency splitting of the proposed sensing structure is also related to κ. κ is an important parameter for implementing an ES, which can also enhance the frequency splitting. Therefore, compared with the previous work, the proposed sensing structure exhibits a larger frequency splitting enhancement effect, which is jointly determined by the performance of an ES and the Sagnac–Fizeau shift. When Ω=2 kHz, the sensing structure works near an ES and the two eigenvalues are no longer degenerate, but the frequency splitting still shows nonlinear changes with the perturbation G, as shown in the blue solid line in Figure 2. When Ω=5 kHz, the sensing structure has moved away from an ES. The frequency splitting presents a linear change with the perturbation G, and the eigenvalue splitting further increases (the red dashed line in Figure 2).

As a self-reference sensing mechanism, frequency splitting has the advantage of noise immunity. To compare the frequency splitting between the proposed sensing structure and the traditional ES-based sensing structure, according to Equations (6) and (7), the enhancement factor is defined as

(8) $$\chi =\frac{\Delta \omega }{{\Delta \omega }_{\mathrm{ES}}}=\sqrt{1+\frac{{\Delta \omega }_{\mathrm{sag}}^{2}}{G\kappa +G^{2}}}$$

**Figure 3:**
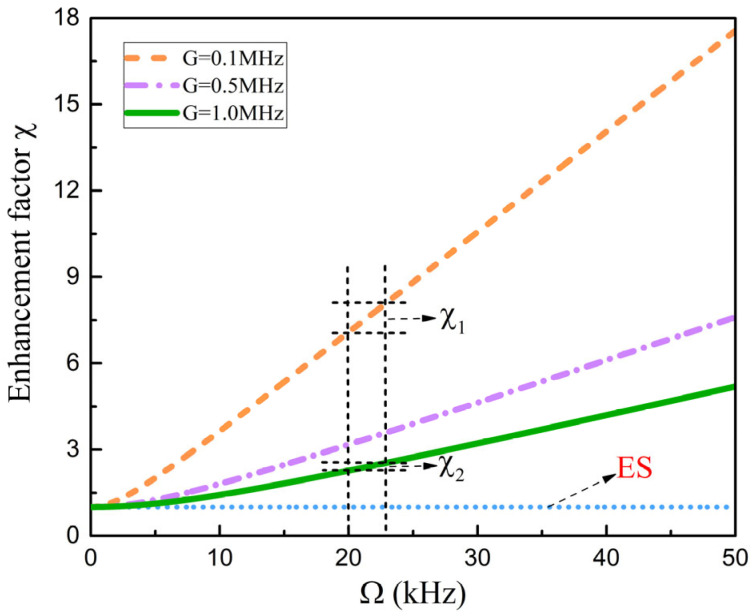
Variation in frequency splitting enhancement factor with angular velocity under different perturbations. The dashed blue line represents that the frequency splitting of the nanoparticle sensing structure based on an ES does not change with Ω. The proposed sensing structure exhibits varying degrees of frequency splitting enhancement with Ω.

As can be seen from Equation (8), when κ is fixed, the enhancement factor depends on Ω and G. The variation in the enhancement factor with Ω under different perturbations is studied (Figure 3). When Ω reaches a certain threshold, the enhancement factor will increase significantly. The reason for the existence of the threshold is the competition between the value of Ω and the value of G, which is ignored in Equation (8) when Ω is small, thus canceling out the enhancement. The optical microcavity has experimentally achieved an Ω of 6 kHz [28], which can reach the threshold value of Ω. On the other hand, the smaller the value of G, the smaller the threshold value of Ω, and the more obvious the enhancement effect of Ω, resulting in a larger enhancement factor, such as $\chi_{1}>\chi_{2}$. Similarly to the characteristics of the ES, this feature helps to improve the minimum detection limit of the nanoparticle.

### 3.2. Manipulating Spectral Line Shape in the ES-Based Structure

**Figure 4:**
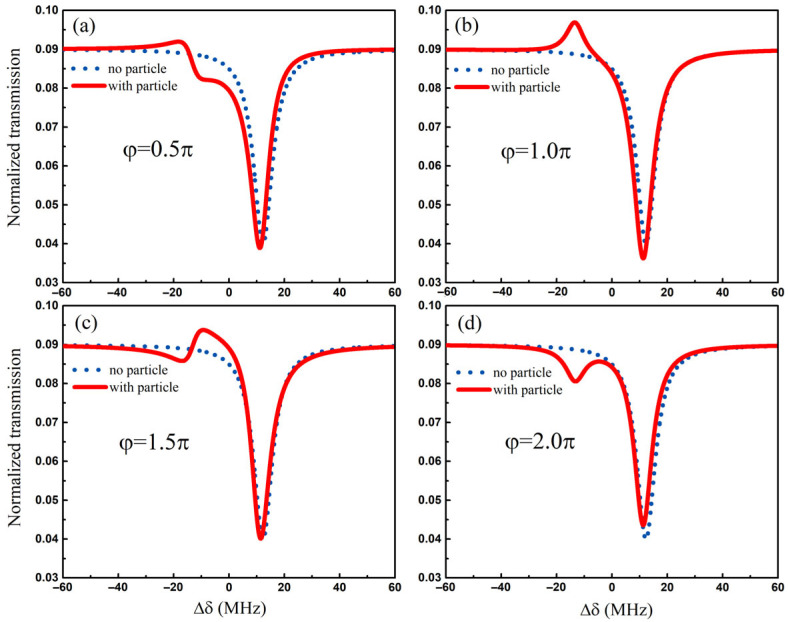
Dependence of the spectral line shape on the phase. (**a**) the phase is 0.5π; (**b**) the phase is 1.0π; (**c**) the phase is 1.5π; (**d**) the phase is 2.0π. The dashed blue line represents that the transmission spectrum *T* is a function of optical detuning Δδ for G=0 MHz. The solid red line indicates that the transmission spectrum *T* is a function of optical detuning Δδ for G=1 MHz. The parameters are Ω=50 kHz.

The size information of a nanoparticle can be obtained by the change in the peak value in the transmission spectrum. By substituting the frequency splitting extracted from the transmission spectrum into Equation (7), the perturbation G of a nanoparticle can be derived. According to g=−Reαf(r→)2ωc/2Vm and $\alpha =4\pi R^{3}\varepsilon_{l}\left(\varepsilon_{p}-\varepsilon_{l}\right)/\left(\varepsilon_{p}+2\varepsilon_{l}\right)$, the size information of a nanoparticle can be obtained in the actual detection scenario. So it is necessary to further investigate the relationship between the evolution of the spectral line shape and the phase φ in the reflection loop. Next, we simulated the transmission spectrum $T={\left|s_{\mathrm{out}}/s_{\mathrm{in}}\right|}^{2}$ under different phases. Figure 4 shows the normalized transmission spectrum with the presence of a nanoparticle under different phases (solid red line) and the transmission spectrum without the presence of a nanoparticle as a contrast (dashed blue line). According to Equation (3), the eigenvalues of the structure are always non-degenerate under Ω≠0. In the absence of a nanoparticle, a counterintuitive single peak without linewidth broadening appears in the transmission spectrum (blue dashed line), even if the phase φ changes. The reason is that two eigenfrequencies exist in the form of bright mode and dark mode states, respectively [29,32]. The bright mode and the dark mode are defined by whether the intrinsic eigenfrequencies of the sensing structure can be observed as peaks in the transmission spectrum. The eigenfrequency in the form of the dark mode is not visible in the transmission spectrum under the unidirectional coupling state. It is only visible after the presence of a nanoparticle causes a reciprocal coupling between the two modes, which makes the dark mode appear in the transmission spectrum. The existence of a phase φ provides conditions for Fano interference between modes. It is worth noting that the Fano line shape exhibits different changes in the phase range 0:2π. As can be seen in Figure 4a, the phase φ=0.5π, and the spectral line shape on the left side of the transmission spectrum is a standard Fano peak, with the two extrema of the Fano profile, i.e., one minimum and one maximum. The standard Fano line shape at the phase φ=1.5π appears in the opposite form, as shown in Figure 4c. For the phase φ=1.0π, the Fano line shape presents a spectrum similar to electromagnetic-induced transparency in Figure 4b. When the phase is 2π, Figure 4d shows a standard dip that is similar to electromagnetically induced absorption. The free regulation of the Fano line shape provides a way to exhibit easily distinguishable frequency splitting in the transmission spectrum.

### 3.3. Enhancement of Frequency Splitting in the Spectrum

**Figure 5:**
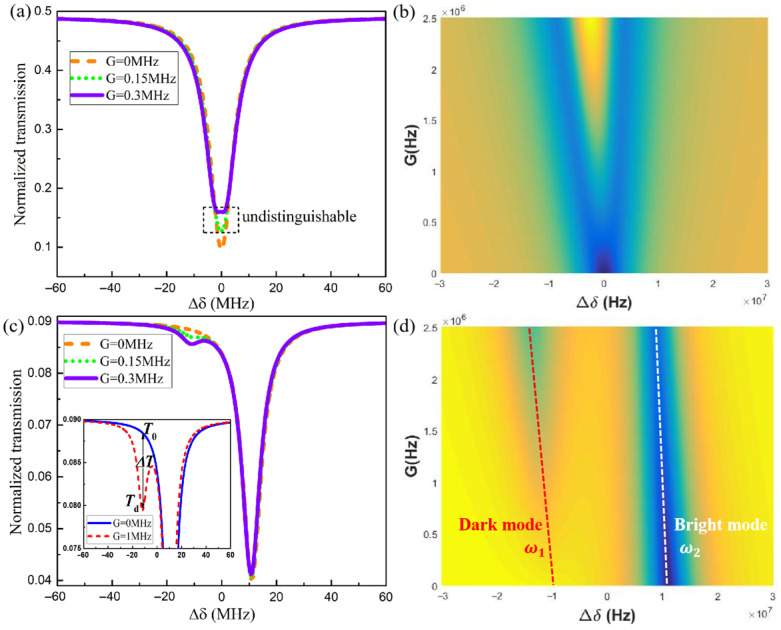
(**a**) Transmission spectrum of the traditional ES-based sensing structure (Ω=0, i.e., a stationary microcavity) for different perturbations. (**b**) Trajectories of transmission peaks in (**a**) as a function of perturbation G. (**c**) Transmission spectrum of the proposed sensing structure (Ω=45kHz, i.e., a spinning microcavity) for different perturbations. (**d**) Trajectories of transmission peaks in (**c**) as a function of perturbation G. The dotted red line corresponds to the dark mode eigenfrequency. And the dotted white line corresponds to the bright mode eigenfrequency.

The size information of a nanoparticle can be obtained by the frequency splitting in the transmission spectrum. Thus, the frequency splitting in the transmission spectrum of the two sensing structures is further analyzed. When Ω=0, Equation (2) can be transformed into the mode evolution of the ES-based structure under non-rotating conditions. Based on the input–output relationship, the transmission spectrum of the conventional ES-based structure is derived. In Figure 5a, there is a single peak with linewidth broadening in the transmission spectrum of the ES-based structure when the perturbation G is less than 0.3 MHz. This is due to the linewidth widening canceling out the frequency splitting. It is generally believed that the splitting phenomenon could be visible only when the eigenvalue splitting is greater than the mode linewidth. At the same time, trajectories of the transmission peaks of the ES-based structure as a function of perturbation G are shown in Figure 5b. The proposed sensing structure is a standard single peak (orange dashed line) at the perturbation G=0 in Figure 5c. With the increase in the perturbation G, the dark mode peak appears gradually. When the perturbation G=0.3 MHz, the proposed sensing structure has an obvious peak splitting phenomenon. It can be seen that the proposed nanoparticle sensing mechanism has an advantage in terms of monitoring peak separation in the transmission spectrum. This is due to the introduction of the Sagnac–Fizeau shift; the dark mode is far away from the bright mode, avoiding the limitation of mode linewidth. Further, we study the change in peak intensity of dark mode intensity caused by the perturbation G, which is defined as $\Delta T=T_{d}-T_{0}$. There is a positive relationship between dark mode intensity ΔT and the perturbation G, as shown in Figure 5d. The dark mode intensity can also be used as a nanoparticle sensing mechanism.

**Figure 6:**
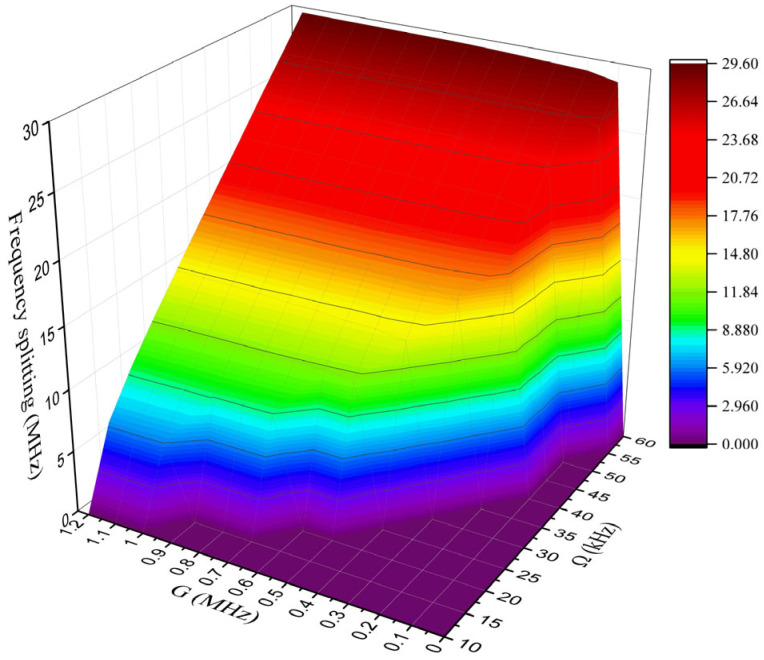
The frequency splitting in the transmission spectrum with angular velocity under different perturbations. When the frequency splitting is zero, it indicates that there is no distinguishable frequency splitting in the transmission spectrum of the proposed sensing structure.

Next, the effect of the perturbation G and the angular velocity Ω on the frequency splitting responses in the transmission spectrum are analyzed. Within the angular velocity Ω range 10~60 kHz, the resolvable frequency splitting value can be extracted directly from the transmission spectrum $T={\left|s_{\mathrm{out}}/s_{\mathrm{in}}\right|}^{2}$. The relevant simulation results are presented in Figure 6. For an Ω of 10 kHz, the smallest detectable nanoparticle perturbation is 1.01 MHz. With the increase in the Ω, the frequency splitting is enhanced in the transmission spectrum. When the Ω reaches 60 kHz, the frequency splitting value reaches 29.3 MHz, and the smallest detectable nanoparticle perturbation is reduced to 0.05 MHz.

### 3.4. Improvement of the Detection Limit

Whether frequency splitting enhancement can improve the performance of the sensor needs to be studied by determining the minimum detection limit. In the actual detection process, the influence of sensor noise should be considered. The signal-to-noise ratio (SNR) of frequency splitting directly determines the minimum detection limit of the nanoparticle sensor. The noise source mainly comes from optical shot noise, electrical shot noise, detector noise, laser relative-intensity noise, laser-frequency noise, etc. [33]. The implementation of the balanced-detection technique and the high-Q-factor microcavity can effectively suppress some noise components. Therefore, the total noise is given by

(9) $$\sigma =\sqrt{\sigma_{\mathrm{DN}}^{2}+\sigma_{\mathrm{OSN}}^{2}+\sigma_{\mathrm{ESN}}^{2}}=\sqrt{\sigma_{\mathrm{DN}}^{2}+2\cdot \left(\hbar \omega +q/\rho \right)\cdot P_{\mathrm{out}}}$$

where $\sigma_{\mathrm{DN}}^{2}$ represents the detector noise, $\sigma_{\mathrm{OSN}}^{2}$ is the optical shot noise, $\sigma_{\mathrm{ESN}}^{2}$ is the electrical shot noise, hω represents the photon energy, q is the electron charge, ρ denotes the detector responsivity, and $P_{\mathrm{out}}$ is the power change of the detector.

The distinguishable frequency splitting in the transmission spectrum occurs after the appearance of the dark mode peak, and the change in the dark mode intensity $\Delta T_{d}$ will lead to the power change of the detector $\Delta P_{\mathrm{out}}=\Delta T_{d}\cdot P_{\mathrm{in}}$. The signal-to-noise ratio is written as [29]

(10) $$\mathrm{SNR}=\Delta P_{\mathrm{out}}/\sigma =\Delta T\cdot P_{\mathrm{in}}/\sqrt{2\left(h\omega +q/\rho \right)\cdot T_{d}\cdot P_{\mathrm{in}}+\sigma_{\mathrm{DN}}^{2}}$$

**Figure 7:**
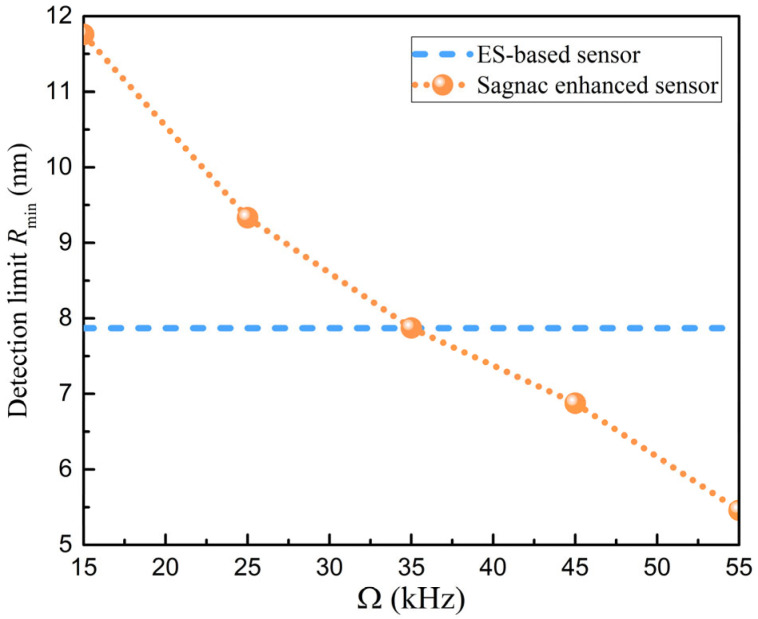
Relationship between the radius of the minimum detectable nanoparticle and the angular velocity. Here, $V_{m}\sim 500\;{\mu m}^{3}$ for a polystyrene (PS) nanoparticle with $\varepsilon_{p}=1.5922^{2}$, $\varepsilon_{l}=1$ for air, and an input power $P_{\mathrm{in}}=100\;\mu W$.

Only when the SNR is greater than 1 can the peak value of the dark mode be detected by the detector, so that the frequency splitting can be resolved in the transmission spectrum to obtain the size information of a nanoparticle. By substituting the values of ΔT for different angular velocities into Equation (10), the minimum detection limit $R_{\min}$ can be obtained. In Figure 7, the detection limit is below Ω=35 kHz, which is less than the minimum detectable limit of the ES-based structure. Because the existence of a small Ω causes the distance between the dark mode and the bright mode, the mode interference is weakened, and the peak intensity is reduced. The line width of the bright mode is limited, so that the frequency splitting is not easy to distinguish. Compared with the ES-based sensing structure, the dark mode is no longer limited by the line width of the bright mode when Ω is greater than 35 kHz. The proposed sensing structure significantly improves the minimum detectable limit of nanoparticle size, and can reach below 5.5 nm at Ω=55 kHz.

### 3.5. Experimental Consideration

Experimentally, in addition to using the balanced detection method to eliminate some optical noise, achieving Sagnac–Fizeau shift in the microcavity based on an ES involves three key points. First of all, it is about how to establish unidirectional coupling between the mode *a*_cw_ and mode *a*_ccw_. According to previous experiments on an ES, unidirectional coupling between the mode *a*_cw_ and mode *a*_ccw_ can be achieved by adding a fiber-optic phase shifter and a partial fiber-optic retroreflector at the right end of the tapered coupled fiber [26,27]. Thus, the sensing structure can initially operate on an ES, and this approach is still applicable. Secondly, the most crucial point is how to achieve coupling stability between the tapered fiber and spinning microcavity in experiments. A spinning microcavity at high angular velocities can cause unnecessary oscillation, which makes it difficult to maintain the gap between the tapered fiber and spinning microcavity within a tolerance range of a few nanometers. The instability of the gap will directly alter the coupling conditions, thereby seriously affecting the performance of the sensing structure. Fluid-film lubrication between the tapered fiber and spinning microcavity provides a simple method for achieving stable coupling which has been experimentally proven [30]. Therefore, we can adopt the method of flying the tapered coupled optical fiber above the rotating microcavity. So, we can use the tapered fiber to fly above the spinning microcavity to achieve coupling stability. Finally, it is necessary to achieve a stable high-Q rotating microcavity at angular velocities up to 55 kHz. When the Q of the microcavity is approximately equal to 10^8^, the loss of the microcavity is mainly dominated by backscattering rather than absorption. Therefore, during the preparation process, gases with higher purity should be used to create microcavities with smooth surfaces.

## 4. Conclusions

In summary, we investigate the application of the Sagnac–Fizeau shift to nanoparticle sensing in an ES-based structure. The frequency splitting enhancement factor is more than one magnitude higher than that of the traditional ES-based sensing structure for weak perturbations. The regulation of the spectral line shape is realized through the phase 0~2π in the reflection loop. Further, the appearance of bright and dark modes under unidirectional coupling overcomes the limitation of mode linewidth, which increases the detectable frequency splitting in the transmission spectrum. Compared with the traditional ES-based sensing structure, the proposed nanoparticle sensing structure makes the minimum detection limit of the nanoparticle less than 5.5 nm. Our proposed scheme provides a feasible way to enhance the performance of a non-Hermitian sensing device.

## Data Availability

Data will be made available on request.

## Appendix A

**Table A1:** List of simulation parameters.

Parameters	Symbol
Diameter of the microcavity	*d*
Input light field	*S*_in_
Output light field	*S*_out_
Wavelength of input light	λ
Coupling coefficient	$\gamma_{\mathrm{ex}}$
Reflection coefficient of the partially reflective mirror	*r*_m_
Transmission coefficient of the partially reflective mirror	*t*_m_
Unidirectional coupling strength	κ
Angular velocity	Ω
Transmission amplitude in the reflection	$t_{0}$
Phase accumulation in the reflection	φ
Refractive index of the microcavity	n
Resonant frequency of a stationary microcavity	$\omega_{c}$
Speed of light	c
Dispersion term	dn/dλ
Total loss of the microcavity	$\gamma_{t}$
Intrinsic loss of the microcavity	$\gamma_{0}$
Complex perturbation induced by the nanoparticle	G
Coupling rate induced by the nanoparticle	g
Polarizability of the nanoparticle	α
Radius of the nanoparticle	R
Relative permittivity of the nanoparticle	$\varepsilon_{p}$
Relative permittivity of the environment medium	$\varepsilon_{l}$
Microcavity mode function	$f_{\left(\vec{r}\right)}^{2}$
Mode volume of the microcavity	$V_{m}$
Loss caused by scattering and absorption of the nanoparticle	$\gamma_{n}$
Detector noise	$\sigma_{\mathrm{DN}}^{2}$
Optical shot noise	$\sigma_{\mathrm{OSN}}^{2}$
Electrical shot noise	$\sigma_{\mathrm{ESN}}^{2}$
Photon energy	hω
Electron charge	q
Detector responsivity	ρ
Input power	$P_{\mathrm{in}}$
Power change in the detector	$P_{\mathrm{out}}$
